# Lake sediments with Azorean tephra reveal ice-free conditions on coastal northwest Spitsbergen during the Last Glacial Maximum

**DOI:** 10.1126/sciadv.aaw5980

**Published:** 2019-10-23

**Authors:** Willem G. M. van der Bilt, Christine S. Lane

**Affiliations:** 1 Department of Earth Science, University of Bergen, Allégaten 41, 5007 Bergen, Norway.; 2 Bjerknes Centre for Climate Research, Bergen, Norway.; 3 Department of Geography, University of Cambridge, Downing Place, CB2 3EN Cambridge, UK.

## Abstract

Lake sediments retrieved from the beds of former nonerosive ice sheets offer unique possibilities to constrain changes in the extent and style of past glaciation, and place them in an absolutely dated context. We present the first pre-Holocene lake sediments from Arctic Svalbard. Radiocarbon dating of terrestrial plant fossils reveals that the investigated catchment was unglaciated and vegetated between 30 and 20 ka B.P. during the global Last Glacial Maximum. The presence of volcanic ash from a contemporaneous Azorean eruption also provides evidence for ice-free conditions. Indicators of sediment compaction and a depositional hiatus suggest subsequent coverage by nonerosive ice until 11 ka B.P. Comparison with regional paleoclimate data indicates that sea ice variability controlled this pattern of ice sheet evolution by modulating moisture supply. Facing rapid regional sea ice losses, our findings have implications for the future response of the Arctic’s cryosphere, a major driver of global sea-level rise.

## Teaser

Lake sediments reveal that parts of the High Arctic were ice free and vegetated during the culmination of the Last Ice Age.

## INTRODUCTION

For decades, the preservation of pre–Last Glacial Maximum (LGM) landforms stirred debate as to whether northwest Spitsbergen on the Svalbard archipelago was ice-covered during the Late Weichselian [30 to 12 calibrated (cal.) ka B.P.] (*1*, *2*). There is now broad consensus that ice extended to the shelf edge and drained through fjords and troughs, while less active ice occupied intermediary areas (*3*, *4*). A wealth of recent cosmogenic nuclide data reveals that this ice sheet was highly dynamic and left a varied imprint on the landscape. Notably, age differences between dated bedrock and boulders suggest that areas were (periodically) covered by nonerosive (cold-based) ice or even exposed during the LGM (*5*–*7*). However, in these terrains, cosmogenic inheritance complicates interpretations about the timing, style, and duration of past glaciation (*8*). Robust chronological control is vital to confidently constrain ice sheet change across time and space. Discoveries from the beds of former nonerosive ice sheets on Baffin Island and Greenland show that Arctic lakes hold great potential to corroborate cosmogenic evidence (*9*, *10*). In these basins, sediments that can be absolutely dated accumulated during ice-free periods, while overriding nonerosive ice preserved and compacted these deposits. This study describes the first pre-Holocene lake sediments reported from Svalbard. We present three bryophyte-derived ^14^C ages, a new regional tephra marker of Azorean provenance, as well as stratigraphic evidence to demonstrate that parts of coastal northwest Spitsbergen were ice free and vegetated from ~30 to 20 cal. ka B.P., before being overrun by nonerosive (cold-based) ice until ~11 cal. ka B.P.

## MATERIALS AND METHODS

### Setting

**Fig. 1:**
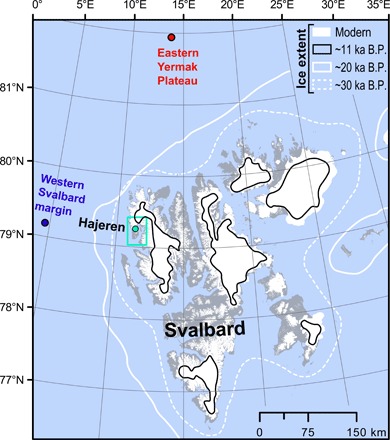
Overview map of the Svalbard archipelago. Our study site, Lake Hajeren, is highlighted with a green dot; the green inset marks the extent of the detailed map in fig. S1. Modern glacier extent is shown, along with (most credible) reconstructed ice margin positions after (*45*) for key intervals: 32 to 30 ka B.P. (referred to as ~30 ka B.P.) (the onset of late Weichselian sedimentation in Hajeren), c. 20 ka B.P. (when the lake was overrun by nonerosive ice), and c. 11 ka B.P. (when lacustrine sedimentation resumed). We also indicate the localities of the sea ice reconstructions from the Yermak plateau and the western Svalbard margin that are shown in Fig. 5 (*47*).

Our study site, Lake Hajeren (79.26°N, 11.52°E), is situated on the Mitra peninsula of northwest Spitsbergen (Fig. 1 and fig. S1). As attested by a combination of minimal isostatic unloading and the preservation of pre-Weichselian landforms or surfaces [e.g., (*3*, *11*)], this area may have harbored ice-free areas during the Late Weichselian. Hajeren covers 0.23 km^2^, has a maximum depth of ~20 m, and sits at an elevation of 35 m above sea level (a.s.l.)—just above the 32 m a.s.l. postglacial marine limit reported in nearby Trongdalen (*3*), ~5 km south (fig. S1A). The catchment measures ~3 km^2^ and comprises large tracts of gently sloping weathered surfaces covered by polygonal ground, solifluction lobes, and overdimensioned channels that drain meltwater from two small cirque glaciers (fig. S1B) (*12*). The alpine terrain of the surrounding mountains and fjords indicates that glacial erosion has modified the local landscape. The orientation of glacial striations and flutes suggests that ice mostly flowed along the southeast-trending adjacent Krossfjord during glacial maxima (fig. S1A) (*3*). Exposure-dated erratics from Trongdalen indicate that ice last retreated from this fjord around 12.2 cal. ka B.P. (*3*). However, local glaciers persisted in the Hajeren catchment and readvanced around 9.5 cal. ka B.P. before disappearing after 7 cal. ka B.P.: The small glaciers that presently occupy two cirques reformed during the Late Holocene (*13*). The bedrock lithologies that underlie both catchment and upstream ice drainage basins comprise schist or migmatite (*14*). Two sediment sequences were extracted from Lake Hajeren with a piston corer during fieldwork in 2012, only one of which contained undisturbed sediments (core HAP0212) (*13*).

### Sediment stratigraphy

This study focuses on HAP0212, a 332-cm-long undisturbed core taken from the deepest part of the basin. Here, we reappraised bottom unit 4 (272 to 320 cm)— homogenous, dense, and minerogenic sediments that were excluded from previous work on the Holocene lake history by (*13*) because of “improbably old” radiocarbon ages [16,580 ± 180 and 25,100 ± 300 ^14^C yr B.P. (radiocarbon years before the present)] and signs of dewatering. We characterized the stratigraphy of unit 4 sediments using four parameters on a common 0.5-cm resolution. Sediment density [dry bulk density (DBD)] and water content, basic measures for compaction (*9*), were determined. Magnetic susceptibility (MS), an indicator of detrital input (*15*), was measured using a Bartington MS2E sensor. We also measured loss on ignition (LOI) on these samples, a basic measure of organic content, following the protocol by (*16*). To assess sediment provenance, we carried out x-ray diffraction (XRD) analysis on ^14^C-dated depth intervals (*n* = 3). For this purpose, we sieved sample material through a 63-μm mesh and separated clay from silt using gravity settling. Both fine fractions were then analyzed as randomly oriented dry powders on a Bruker D8 ADVANCE ECO X-ray diffractometer. Last, we visualized sedimentary structure in three dimensions (3-D) using a ProCon X-Ray ALPHA Computed Tomography (CT) scanner. Extensive sampling of HAP0212 for previous work (*13*) has restricted this exercise to selected depth intervals. In addition, we tried to extract lipids from analyzed unit 4 sediments in mixture of dichloromethane (DCM) and methanol (9:1 volume %) with a Dionex ASE 350 system. So far, all analyzed samples (*n* = 5) were devoid of identifiable compounds following gas chromatography mass spectrometry and flame ionization detection.

### Tephra analyses

Volcanic glass (tephra) was extracted from the investigated sediments using the flotation procedure of (*17*) by sieving at 15 μm followed by density extraction using different densities of sodium polytungstate heavy liquid (1.95 to 2.55 g/cm^3^). To detect discrete horizons, we sampled and analyzed the entire length of targeted unit 4 sediments. First, we focused on contiguous 10-cm sediment slices. Then, we zoomed in on sections that contained glass using 1-cm interval samples. Last, shards were picked for analysis from the discrete tephra horizon presented here (278.5-cm core depth) with the help of a gas chromatography syringe [cf. (*18*)]. Individual tephra shards were then geochemically characterized using wavelength electron microprobe analysis to measure major and minor element oxide concentrations. Analyses were carried out at the Electron Probe Microanalysis Facility at the School of Geosciences of the University of Edinburgh. For this purpose, we used a Cameca SX100 instrument that was operated at an accelerating voltage of 15 kV, with variable beam currents of 0.5 nA (Na/Al), 2 nA (Mg/Si/K/Fe/Ca), and 60 nA (P/Ti/Mn), and a beam diameter of 6 μm. Secondary glass standards (LIPARI and BCR2g) were analyzed between and within runs to monitor analytical precision (table S1).

### Geochronology

**Fig. 2:**
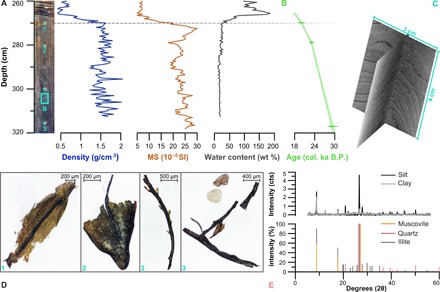
Stratigraphic context of analyzed unit 4 sediments from Lake Hajeren. (**A**) Down-core density, magnetic susceptibility (MS), and water content (compared to dry sediment weight) measurements. (**B**) Clam-calibrated chronology. The core image on the left-hand side marks the sampling locations of the analyzed material shown in the other panels using corresponding numbers or letters. (**C**) Computed tomography (CT) slice that highlights the laminated nature of the investigated sediments. (**D**) Plate(s) of dated plant macrofossil remains from dated horizons. Plates 1 and 3 show representative material from the horizon previously dated by (*13*) (table S2), while material from horizon 2 was dated for this study. (**E**) X-ray diffraction (XRD) diffractograms of the clay (<6 μm) and silt (6 to 63 μm) fraction of unit 4 sediments, plotted together with reference stick patterns of the most abundant minerals—muscovite, quartz, and illite. Photo credit: Willem van der Bilt, University of Bergen.

We constrained the age of the investigated core section with three radiocarbon samples of terrestrial plant macrofossils. LuS 10868 (269.5 cm) and LuS 10870 (317 cm), first reported by (*13*) but designated as outliers, constrain the upper and lower sections of unit 4, respectively. For this study, we submitted an additional terrestrial plant macrofossil sample (LuS 13913) to the same laboratory (Lund Radiocarbon Dating Laboratory) from the same depth as the presented tephra isochron (278.5 cm). All ages were calibrated with the IntCal13 curve before obtaining an age-depth model with Clam 2.2 using a linear fit (*19*, *20*). To assess the condition and origin of dated material from all horizons, we used a light microscope fitted with a ZEISS Axiocam 105 camera. All submitted material consists of several (maximum of 10) terrestrial plant macrofossils, identified as bryophytes (mosses, Fig. 2).

## RESULTS

### Late Weichselian lake sediments

The chronology shown in Fig. 2B indicates that Lake Hajeren received sediment input between 29,966 to 28,516 cal. yr B.P. and 20,466 to 19,571 cal. yr B.P. (2σ). The presented ^14^C dates are in stratigraphic order and separated by stable time increments, which suggests that the investigated sediments have neither been disturbed nor reworked (table S2). The CT orthoslices in Fig. 2C support this notion by revealing intact centimeter-scale laminations. As shown by the micrograph plates of Fig. 2D, we were able to pick and date terrestrial macrofossils, providing robust chronological control. The presence of this material indicates that (part of) the Hajeren watershed was ice free and sustained plant life (bryophytes; mosses) during the Late Weichselian.

Complementing the presented chronological evidence, minimal organic content—reflected by stable low (1.5%) LOI values (fig. S2A) (*13*)—helps exclude the possibility that the investigated sediments were deposited during (productive) interglacial conditions. This notion is supported by the absence of lipid biomarker compounds, ubiquitous throughout the Holocene (*21*), analyzed in unit 4 samples. Last, picked and pictured macrofossils exclusively comprise bryophytes (mosses). At present, this phylum only dominates the flora of Antarctica because of their unique ability to withstand extreme cold and complete desiccation (*22*).

Mineralogical (XRD) analyses allay concerns of carbonate contamination of radiocarbon ages by freshwater reservoir effects: unit 4 host sediments that predominantly consist of the phyllosilicates (illite and muscovite) and quartz minerals that dominate the bedrock of the surrounding catchment (Fig. 2E) (*14*). The first ^14^C age above the investigated late Weichselian sediments (unit 4) at 265 cm yields an Early Holocene age of 11,260 to 11,082 cal. yr B.P. (2σ) (*13*), indicating an ~9000-year hiatus. In the absence of a truncated contact with the overlying sediments or other indications of an erosive boundary, we argue that this interval marks a period of nondeposition. On the basis of high undrained shear strength values (126 kPa), van der Bilt and co-workers (*13*) suggest compaction of unit 4. Our findings support this interpretation as downcore measurements reveal that the investigated sediments are consistently dewatered (water content ≈ 20%) and dense (DBD ≈ 1.5 g/cm^3^) (Fig. 2A). Together with high MS (MS ≈ 25 × 10^−5^ SI) values, this depositional signature is notably different from the Holocene sediments in Lake Hajeren (fig. S2A) but characteristic for compression by ice in similar Arctic lakes where pre-Holocene sediments have been preserved (*9*). Under such a scenario, perennial lake ice coverage and overriding nonerosive (cold-based) ice preserve and compress sediments, respectively (*10*).

### Azorean tephra

**Fig. 3:**
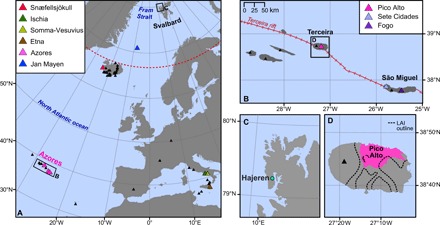
Overview maps with the locations of discussed tephra sites and samples. (**A**) North Atlantic region, delineating insets (B) and (C) with rectangles and marking active volcanoes with black triangles. Discussed eruptive centers are shown in different colors. (**B**) Azores archipelago, showing the Terceira rift after (*58*) and marking mentioned volcanoes on the islands of São Miguel and Terceira. (**C**) The Lake Hajeren study site and northwest Spitsbergen. (**D**) Terceira Island, delimiting Lajes-Angra Ignimbrite (LAI) deposits from the Pico Alto volcano (*25*).

**Fig. 4:**
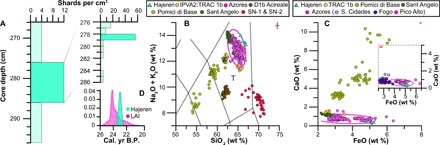
Tephra concentrations and geochemistry. (**A**) Shard counts in the analyzed horizon (9539) and adjacent 1- and 10-cm core intervals (table S3). (**B**) Total alkali-silica (TAS) diagram, comparing the normalized geochemical composition of analyzed shards from Lake Hajeren with reference glass material from Trachytic (T) volcanic sources and eruptions from North Atlantic and Mediterranean sources. (**C**) FeO(*t*) versus CaO biplot to distinguish Azorean tephras from other sources and one another (inset) after (*28*, *32*). Inset red crosses visualize analytical uncertainty based on secondary standard measurements. Ellipses encapsulate 95% of plotted reference material from the Azores (Fig. 3B) and Pico Alto from Terceira (Fig. 3C). Table S4 lists data sources. (**D**) Calibrated radiocarbon ages of the analyzed tephra horizon from the Lake Hajeren study site and the Summed Probability Distribution of published dates (*n* = 3) at the base of the LAI Ignimbrite (table S5) (*25*, *39*).

Our tephra data provide additional evidence for ice-free conditions in the Hajeren watershed during the Late Weichselian. We located a distinct cryptotephra layer between 278- and 279-cm core depth (Fig. 4A), composed of clear glass shards with open vesicle cuspate forms, ~20 μm in size. A discrete shard maximum (56 g/cm^3^) suggests that ash was deposited from primary air fall (*23*), allaying concerns about reworking such as delayed release from snow or ice. This notion is supported by a homogeneous geochemistry; secondary deposition typically mixes tephra from different eruptions (*24*). Major and minor oxide data from the four diminutive shards that were analyzed following three rounds of extraction reveal a trachytic composition, with normalized SiO_2_ weight percentages between 65.62 and 66.8% and a total alkali (Na_2_O + K_2_O) content ranging from 13.29 to 14% (table S6). To find a geochemical match, we compared our data to reference material of trachytic eruptions from volcanoes that were active around the time of deposition (Fig. 3A and table S4). We delimit this interval between 19 and 23 cal. ka B.P. by using the age-depth model presented in Fig. 2B, which is constrained at this depth by radiocarbon age LuS 13913 (range, 20,466 to 19,571 cal. yr B.P. 2σ). Possible candidates include the D1b Acireale eruption of Etna, the Pomici di Base eruption of Somma-Vesuvius, the Sant Angelo Tephra from Ischia, and the Lajes-Angra Ignimbrite (LAI) from Pico Alto on the Azorean island of Terceira (Fig. 3B and table S4) (*25*–*28*). Contemporaneous trachytic eruptions from the Fogo and Sete Cidades volcanoes on the Azorean island of São Miguel lack reference glass compositional data (Fig. 3B) (*29*, *30*). We address this issue by using other glass data from these sources (*30*–*33*). Last, we include data from the most proximal sources of trachyte tephra, Jan Mayen and Snæfellsjökull on Iceland. While reported Late Pleistocene and Holocene Jan Mayen eruptives exclusively comprise basanite and trachybasalt (*34*), so little is known about the eruptive history of the island that we include the most recent (580 to 640 cal. ka B.P.) trachyte ash (*35*). From Snæfellsjökull, we include data from the two characterized tephras from this system, produced by the Holocene SN-1 and SN-2 eruptions (*36*, *37*). Low (<1) K_2_O/Na_2_O ratios suggest that the analyzed shards derive from an anorogenic interplate volcanic setting (*38*), such as Etna or the Azores; a total alkali-silica (TAS) plot shows the analyzed shards group closest to reference material from the latter source (Fig. 4B). An Azorean provenance is also supported by the bivariate plots of Fig. 4C, which highlight the characteristically low CaO (<1%) content of Azorean tephra, as well as its high FeO(*t*) concentrations (>4.3%) (*28*, *32*).

Comparison with available data from Azorean eruptions indicates that our samples derive from the island of Terceira (Fig. 4C, inset). Here, the Pico Alto volcano produced the largest pyroclastic deposit on the island (3.3 km^3^ dense rock equivalent) during the LAI eruption, which has been dated to between ~20 and 23 ^14^C ka B.P. (*25*, *39*). The first stage of this cataclysmic event produced fine ash with a high potential for distal dispersal (*40*). We used aforementioned published ^14^C ages (*n* = 3) taken from the base of LAI deposits to constrain the onset of this phase (table S5) (*25*, *39*). Timing of LAI ash dispersal (range, 22,711 to 24,536 cal. yr B.P. 2σ) overlaps with the age distribution of the presented tephra isochron (Fig. 4D), providing chronological evidence in support of correlation. We should note that no other Pico Alto eruption occurred during this period; a preceding Ignimbrite-forming event has been dated to ~35 ka B.P. (*25*). Moreover, glass from the other active volcanic center on Terceira (Santa Bárbara; Fig. 3C) has a distinctly different (nonperalkaline) geochemistry (*41*). Our finding marks the first find of LAI ash outside Terceira, highlights its potential pan-North Atlantic LGM isochron, and expands the known dispersal distance of Azorean tephra by thousands of kilometers.

## DISCUSSION

### Glacio-climatic conditions on northwest Spitsbergen during the Late Weichselian

The presented chronostratigraphic framework demonstrates that parts of the coastal northwest Spitsbergen remained unglaciated between 30 and 20 ka B.P. before being overrun by nonerosive (cold-based) ice. The inferred timing of this transition from ice-free to ice-covered around 20 cal. ka B.P. is consistent with the most regional evidence of the culmination of ice sheet expansion. Using amino acid ratios on dated shells, Mangerud and co-workers (*42*) argue that ice overrode the west coast of Spitsbergen after 22 cal. ka B.P. and advanced toward the shelf edge between 23 and 21 cal. ka B.P., as indicated by debris lobe deposition along the western Svalbard margin [e.g., (*43*, *44*)]. We should note that the compilation by (*45*) suggests that ice already extended beyond the modern coast ~30 cal. ka B.P. (Fig. 1). However, as stated by the authors in their supplementary database, this interpretation is based on sparse evidence or cosmogenic (^10^Be) dates with a complex exposure (inheritance) history. The reported association of undisturbed and compacted sediments with an overlying hiatus in Lake Hajeren is indicative for coverage by nonerosive (cold-based) ice, as observed in similar settings elsewhere [e.g., (*10*)]. Our data thus support the notion that fast-flowing ice was restricted to troughs and fjords during the Late Weichselian (*4*). Basal conditions need to remain at or below the pressure melting point to maintain cold-based glaciation. This restricts the possible thickness of the Late Weichselian ice over low-lying Lake Hajeren, as also suggested by minimum ice surface estimates (<300 m a.s.l.) based on exposure dates derived from the surrounding Mitra peninsula (fig. S1B) (*3*). Because nunataks may have remained unglaciated throughout the Late Weichselian (*46*), plants like the bryophytes (mosses) pictured and dated for this study could have survived on higher ground after ice overrode coastal lowlands.

**Fig. 5:**
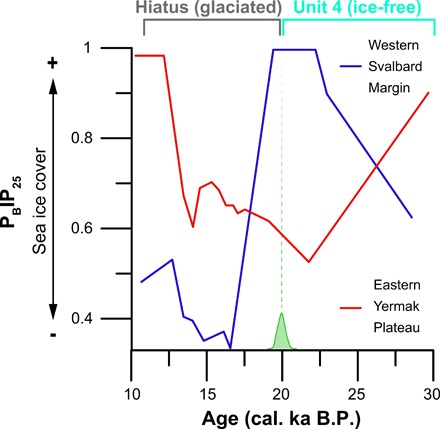
P_B_IP_25_-derived changes in seasonal sea ice conditions during the discussed time interval of 30 to 11 ka B.P. from the western Svalbard margin (core PS93/006-1) and eastern Yermak plateau (core PS92/039-2) after (*12*). The time spans covered by analyzed unit 4 in core HAP0212 and the overlying hiatus are highlighted, while we show the calibrated range of the ^14^C age (LuS 10868, green bell curve; also see table S2) that separates both intervals.

From a paleoclimate perspective, regional marine geological records reveal that the advance of ice over Hajeren and across Svalbard around 20 cal. ka B.P. coincides with sea ice shifts of an unprecedented magnitude during the discussed ~30 to 11 cal. ka B.P. While perennial sea ice formed along the western Svalbard margin after ~22.5 cal. ka B.P., seasonally open conditions were established farther north on the eastern Yermak plateau (Figs. 1 and 5) (*47*, *48*). Kremer and co-workers (*49*) argue that katabatic winds originating from the expanding Barents Ice Sheet played a critical role in creating open water conditions at this time of minimal summer insolation and low surface temperatures. Knies and co-workers (*50*) argue that wind-driven upwelling of mild Atlantic waters, which continued to flow to Svalbard below the surface, helped prevent sea ice formation. In general, sea ice coverage blocks the transfer of moisture from ocean to atmosphere (*51*); the existence of seasonally open water therefore greatly enhances surface moisture fluxes. Hebbeln and co-workers (*52*) suggest that the availability of moisture was a critical constraint for the buildup of ice on Svalbard, a polar desert at the time. Proxy-constrained modeling experiments show that ice sheet expansion toward reconstructed LGM limits on Svalbard required a 130% increase in precipitation from present-day (inter-glacial) conditions (*53*). At around 20 cal. ka B.P., prevalent northeasterly polar winds could pick up extra moisture from the seasonally open waters of the Yermak plateau before releasing it as snow over northwest Spitsbergen—the first landmass in their track (Fig. 1). Conversely, perennial sea ice coverage on the Yermak plateau from ~30 to 20 cal. ka B.P. limited the supply of precipitation to our study area (*47*), restricting ice buildup (*47*). The dominance of bryophytes (Fig. 2D), which can survive complete desiccation (*22*), also supports extreme aridity during this period. Still, there is ample evidence that ice domes occupied the interior of Spitsbergen at the time (*5*, *7*). We, however, argue that the ice streams bordering the surrounding Mitra peninsula drained ice away from the Hajeren catchment (fig. S1A).

Resumption of lacustrine sedimentation around 11 cal. ka B.P. provides a minimal age for the deglaciation of the Hajeren catchment (fig. S2B). This estimate is in agreement with geochronological evidence from the surrounding Mitra peninsula (12.2 cal. ka B.P.) (*3*), other parts of northwest Spitsbergen (~14 cal. ka B.P.) (*7*), and the wider Svalbard archipelago (11 cal. ka B.P.) (Fig. 1) (*11*). Climatologically, deglaciation can be attributed to high radiative forcing and intensifying heat advection by the surface ocean (*54*).

## CONCLUSIONS

We report the first evidence of pre-Holocene lake sediments on the Arctic Svalbard archipelago. The presented chronostratigraphic data reveal that unglaciated areas existed on northwest Spitsbergen between 30 and 20 cal. ka B.P., coincident with the LGM. Bryophyte macrofossils, used for dating, indicate that these cryptic refugia were vegetated. In light of evidence for ice-free conditions on high (>300 m a.s.l.) plateaus since 80 cal. ka B.P. (*46*), these findings raise the possibility that plants may have endured the Last Glacial period on Svalbard. The analyzed sediments were preserved in a lowland basin and thus confirm recent suggestions that even low-lying areas on Svalbard were covered by nonerosive ice during the LGM (*6*). Similar sites are ubiquitous on Svalbard, raising the possibility that more and possibly older sequences may be retrieved in the future. Comparison with regional paleoclimate records suggests that the culmination of ice expansion around 20 cal. ka B.P. was triggered by enhanced moisture fluxes from seasonally open waters upwind (northeast) from the Lake Hajeren site. This interpretation underscores the importance of hydrological change for the evolution of Arctic ice sheets, which face a future that is warmer as well as wetter (*55*). The proposed mechanism may also explain the similarly late LGM culmination of precipitation-starved ice sheets on Greenland and Ellesmere Island (*56*, *57*). Last, the reported tephra isochron marks the most distal find of volcanic ash from the Azores and represents a valuable new LGM time marker that has the potential to synchronize paleoclimate reconstructions across the entire North Atlantic region.

## Supplementary Material

http://advances.sciencemag.org/cgi/content/full/5/10/eaaw5980/DC1

Download PDF

Lake sediments with Azorean tephra reveal ice-free conditions on coastal northwest Spitsbergen during the Last Glacial Maximum

## SUPPLEMENTARY MATERIALS

Supplementary material for this article is available at http://advances.sciencemag.org/cgi/content/full/5/10/eaaw5980/DC1 Fig. S1. Overview maps of our study area and site. Fig. S2. The full stratigraphy and chronology of investigated core HAP0212. Table S1. Major and minor oxide data of glass standards, along with calculated means and (weighted) SDs (2σ) of replicate measurements. Table S2. Overview of presented radiocarbon (^14^C) samples. Table S3. Glass (tephra) shard counts in 10-cm slices of core HAP0212, as well as 1-cm resolution counts for the selected 276.5- to 285.5-cm interval shown in Fig. 4A. Table S4. Published ages and reference glass data sources for specific eruptions from particular volcanic sources that are discussed and shown in the main text (Figs. 3 and 4). Table S5. Published radiocarbon ages that were taken from the base of LAI deposit and used to calculate the onset of the eruption (Fig. 4D). Table S6. Major and minor oxide data (normalized), along with basic statistics (including the coefficient of variation), of the analyzed (*n* = 4) tephra shards presented in this study.
