# Supplementary material for: Lake sediments with Azorean tephra reveal ice-free conditions on coastal northwest Spitsbergen during the Last Glacial Maximum
Source: Sci Adv. 2019 Oct 23;5(10):eaaw5980. doi: 10.1126/sciadv.aaw5980 (PMC6810458; doi:10.1126/sciadv.aaw5980)
Supplement: http://advances.sciencemag.org/cgi/content/full/5/10/eaaw5980/DC1 [file supp_5_10_eaaw5980__index.html]

Science Advances | Science AdvancesAAASSearchScience AdvancesMenu

## Supplementary Materials

**This PDF file includes:**

- Fig. S1. Overview maps of our study area and site.
- Fig. S2. The full stratigraphy and chronology of investigated core HAP0212.
- Table S1. Major and minor oxide data of glass standards, along with calculated means and (weighted) SDs (2σ) of replicate measurements.
- Table S2. Overview of presented radiocarbon (14C) samples.
- Table S3. Glass (tephra) shard counts in 10-cm slices of core HAP0212, as well as 1-cm resolution counts for the selected 276.5- to 285.5-cm interval shown in Fig. 4A.
- Table S4. Published ages and reference glass data sources for specific eruptions from particular volcanic sources that are discussed and shown in the main text (Figs. 3 and 4).
- Table S5. Published radiocarbon ages that were taken from the base of LAI deposit and used to calculate the onset of the eruption (Fig. 4D).
- Table S6. Major and minor oxide data (normalized), along with basic statistics (including the coefficient of variation), of the analyzed (*n* = 4) tephra shards presented in this study.

Download PDF

**Files in this Data Supplement:**

- Adobe PDF - aaw5980\_SM.pdf
