# Supplementary material for: Lake sediments with Azorean tephra reveal ice-free conditions on coastal northwest Spitsbergen during the Last Glacial Maximum
Source: Sci Adv. 2019 Oct 23;5(10):eaaw5980. doi: 10.1126/sciadv.aaw5980 (PMC6810458; doi:10.1126/sciadv.aaw5980)
Supplement: Download PDF [file aaw5980_SM.pdf]

Science Advances
AAAS

advances.sciencemag.org/cgi/content/full/5/10/eaaw5980/DC1

Supplementary Materials for

Lake sediments with Azorean tephra reveal ice-free conditions on coastal northwest Spitsbergen during the Last Glacial Maximum

Willem G. M. van der Bilt* and Christine S. Lane

*Corresponding author. Email: willemvanderbilt@uib.no

Published 23 October 2019, *Sci. Adv.* **5**, eaaw5980 (2018)
DOI: 10.1126/sciadv.aaw5980

This PDF file includes:

Fig. S1. Overview maps of our study area and site.
Fig. S2. The full stratigraphy and chronology of investigated core HAP0212.
Table S1. Major and minor oxide data of glass standards, along with calculated means and (weighted) SDs (2σ) of replicate measurements.
Table S2. Overview of presented radiocarbon (^{14}C) samples.
Table S3. Glass (tephra) shard counts in 10-cm slices of core HAP0212, as well as 1-cm resolution counts for the selected 276.5- to 285.5-cm interval shown in Fig. 4A.
Table S4. Published ages and reference glass data sources for specific eruptions from particular volcanic sources that are discussed and shown in the main text (Figs. 3 and 4).
Table S5. Published radiocarbon ages that were taken from the base of LAI deposit and used to calculate the onset of the eruption (Fig. 4D).
Table S6. Major and minor oxide data (normalized), along with basic statistics (including the coefficient of variation), of the analyzed (n = 4) tephra shards presented in this study.

Supplementary Materials

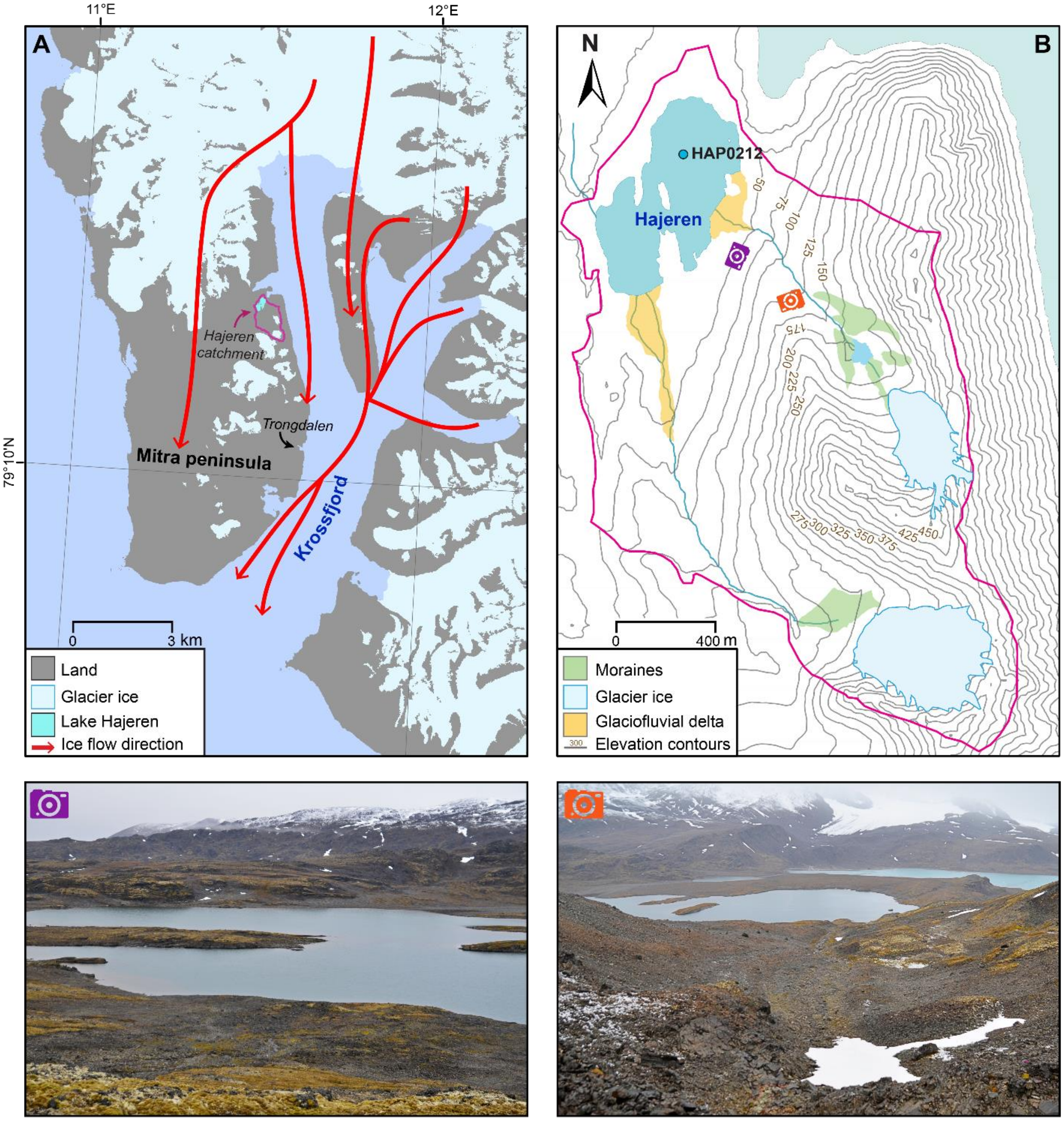

Fig. S1. Overview maps of our study area and site. A: the investigated Hajeren catchment (delineated in pink), nearby Trongdalen (3), the surrounding Mitra peninsula and the adjacent Krossfjord. Ice flow directions were modified from (5) and represents the Late Weichselian timeframe of this study. B: A close-up of the Hajeren catchment, highlighting the topographical barrier that separates the lake from the adjacent Krossfjord, and showing key landforms (sources) along the catchment sediment cascade between glaciers and lake (13). We also show two overview photos of the catchment with symbols that mark their location and angle (corresponding colors). (Photo Credit: Willem van der Bilt, University of Bergen).

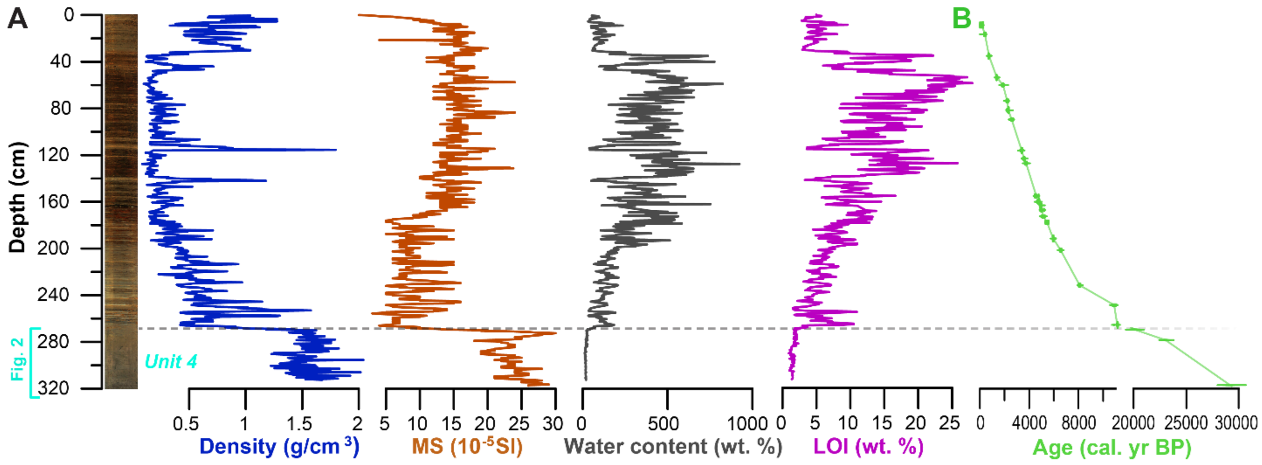

Fig. S2. The full stratigraphy and chronology of investigated core HAP0212. A: Placing Fig. 2 (unit 4) of the main manuscript in a full Holocene context by showing down-core measurements of density, Magnetic Susceptibility (MS), water content (compared to dry sediment weight) and Loss on Ignition (LOI) from the entire length of core HAP0212. B: age-depth models for core HAP0212. The Holocene section is modified after (14).

Table S1. Major and minor oxide data of glass standards, along with calculated means and (weighted) SDs (2σ) of replicate measurements.

				element oxide data - weight percent (%)- mean and 2σ										
**standard**	**n**	**Instrument**	**analysed**	**SiO_2**	**TiO_2**	**$Al2O_3$**	**FeO**	**MnO**	**MgO**	**CaO**	**Na_2O**	**K_2O**	**P_2O_5**	**Total**
LIPARI	13	Cameca SX100	04.03.2016	74.10 (1.19)	0.08 (0.01)	12.92 (0.86)	1.58 (0.27)	0.07 (0.02)	0.05 (0.02)	0.79 (0.13)	4.00 (0.44)	5.12 (0.14)	0.01 (0.01)	98.71
BCR2g	5	Cameca SX100	04.03.2016	51.41 (0.56)	2.27 (0.02)	13.63 (0.60)	12.50 (0.39)	0.2 (0.03)	3.61 (0.11)	7.17 (0.19)	3.31 (0.38)	1.80 (0.11)	0.35 (0.03)	99.26
weighted two standard deviation (2σ)				*1.02*	*0.01*	*0.79*	*0.30*	*0.02*	*0.05*	*0.87*	*0.42*	*0.13*	*0.02*	

Table S2. Overview of presented radiocarbon (^{14}C) samples. *: the depth of this previously published dated sample was accidentally switched with that of LuS 10869 by (14).

**ID**	**depth (cm)**	**Age (^{14}C yr)**	**analytical uncertainty (yr)**	**calibrated age (cal. yr; 2σ)**
LuS 10868	*269.5	16580	180	20466-19571
LuS 13913	278	19090	150	23432-22588
LuS 10870	317	25100	300	29966-28516

Table S3. Glass (tephra) shard counts in 10-cm slices of core HAP0212, as well as 1-cm resolution counts for the selected 276.5- to 285.5-cm interval shown in Fig. 4A.

10cm intervals	Depth (cm)	Shards	Shards (cm^3)	*1cm intervals*	Depth (cm)	Shards	Shards (cm^3)
	171	0	0		276.5	4	16
	181	0	0		277.5	2	8
	191	0	0		278.5	14	56
	201	1	2		279.5	1	4
	211	5	10		280.5	1	4
	221	4	8		281.5	1	4
	231	1	2		282.5	0	0
	241	2	4		283.5	0	0
	251	5	10		284.5	1	4
	261	2	4		285.5	1	4
	271	2	4				
	281	6	12				
	291	2	4				
	301	0	0				
	311	0	0				

Table S4. Published ages and reference glass data sources for specific eruptions from particular volcanic sources that are discussed and shown in the main text (Figs. 3 and 4). Reference numbers correspond to the reference list of the main text.

Volcanic source *(location)*	Eruption	Published age	Reference (glass data)
Sør-Jan (Jan Mayen)	IPVA2: TRAC 1b	580-640 ka BP	(37)
Snæfellsjökull (Iceland)	SN-1	1855 ± 25 cal. yr BP	(38)
	SN-2	3960 ± 100 cal. yr BP	(39)
Etna (Sicily, Italy)	D1b Acireale	19502 ± 302 yr BP	(28)
Somma-Vesuvius (Italy)	Pomici di Base	18220 ± 140 ^{14}C yr BP	(30)
Ischia (Italy)	Sant Angelo	20 ka BP	(30)
Pico Alto (Terceira, Azores)	Lajes–Angra Ignimbrite	20-23 ka BP	(30,25,29)
Fogo (São Miguel, Azores)	Ribeira Chã	14880 ± 60 cal. yr BP	(32)
	Fogo A	5600 cal. yr BP	(34)
	Fogo 1563 AD	1563 AD	(34)
Sete Cidades (São Miguel, Azores)	Santa Bárbara	15740 ± 200 ^{14}C yr BP	(33,35)
	Sete Cidades 1440 AD	1440 AD	(34)

Table S5. Published radiocarbon ages that were taken from the base of LAI deposit and used to calculate the onset of the eruption (Fig. 4D).

Date	^{14}C age	Error	Source
Birm-395	19680	330	(41)
Birm-396	18600	650	(41)
TER03-1A	20110	470	(25)
TER03-1B	20110	110	(25)

Table S6. Major and minor oxide data (normalized), along with basic statistics (including the coefficient of variation), of the analyzed (*n* = 4) tephra shards presented in this study.

				element oxide data - weight percent (%)										
**sample**	**analysis**	**instrument**	**analysis**	**SiO_2**	**TiO_2**	**Al2O 3**	**FeO**	**MnO**	**MgO**	**CaO**	**Na_2O**	**K_2O**	**P_2O_5**	**Total**
9539	10 / 1 .	Cameca SX100	04.03.2016	65.16	0.68	12.64	4.97	0.51	0.23	0.39	8.43	4.73	0.03	97.76
	12 / 1 .	Cameca SX100	04.03.2016	64.68	0.67	12.32	5.04	0.55	0.19	0.39	8.21	4.64	0.04	96.75
	13 / 1 .	Cameca SX100	04.03.2016	63.48	0.67	12.72	4.89	0.54	0.22	0.39	8.39	4.60	0.04	95.94
	14 / 1 .	Cameca SX100	04.03.2016	64.58	0.72	13.40	4.73	0.50	0.23	0.46	9.03	4.76	0.03	98.44
mean (μ)				*64.47*	*0.68*	*12.77*	*4.91*	*0.53*	*0.22*	*0.41*	*8.51*	*4.68*	*0.04*	*97.22*
Standard deviation(σ)				*0.61*	*0.02*	*0.39*	*0.12*	*0.02*	*0.02*	*0.03*	*0.31*	*0.06*	*0.00*	*0.95*
coefficient of variation (cv)				*0.01*	*0.03*	*0.03*	*0.02*	*0.04*	*0.08*	*0.08*	*0.04*	*0.01*	*0.12*	*0.01*
